# The Impact of Environmental Volunteering on Employees, Companies, and Local Communities: A Case Study on Romanian Companies

**DOI:** 10.3390/ijerph21050631

**Published:** 2024-05-16

**Authors:** Silvia Puiu, Mihaela Tinca Udriștioiu

**Affiliations:** 1 Department of Management, Marketing and Business Administration, Faculty of Economics and Business Administration, University of Craiova, 200585 Craiova, Romania; 2 Department of Physics, Faculty of Sciences, University of Craiova, 200585 Craiova, Romania

**Keywords:** environmental volunteering, well-being, productivity, employee, management, community

## Abstract

This paper addresses the importance of organizational environmental volunteering as part of the corporate social responsibility (CSR) strategies of organizations. If more organizations support their employees’ involvement in such projects, there can be hope for mitigating climate change and making the transition to a more sustainable world. We used partial least squares structural equation modeling to analyze the impact of management support on organizational environmental volunteering and of the latter on the employees’ well-being and productivity. Making environmental volunteering part of an organization’s CSR strategy can help the environment, the employees, and the organization itself. The results show a direct and positive relationship between management support and organizational environmental volunteering and between the latter and both the well-being and productivity of employees. The findings will help managers in both public and private organizations to better shape their strategies and encourage their employees to get involved in projects meant to reduce pollution and the carbon footprint.

## 1. Introduction

As climate change has become a reality, and as addressing it becomes increasingly urgent, more and more companies have begun to incorporate CSR programs that are meant to protect the environment by contributing to pollution reduction, promoting renewable energy, and encouraging their employees, customers, and partners to be more eco-friendly in the decisions they make and in their behaviors. The CSR initiatives can focus on some of the sustainable development goals (SDGs) of the 2030 Agenda, such as those related to the climate and people. The programs that support employees in engaging in environmental volunteering are helpful for the planet, the employees’ benefit, and the company’s image as a more responsible organization. Studies show that customers are attracted to companies that adopt more responsible behaviors [1,2,3].

Human resources strategies should consider the importance of organizational environmental volunteering for employees’ well-being, satisfaction, and performance, as well as for attracting them to the company in the first place. As Plewa et al. [4] (p. 643) state, organizational volunteering is “an effective employee engagement initiative”.

As climate change is something that affects us all [5,6,7], we were motivated in our study by a vast amount of literature that shows that environmental volunteering has numerous benefits. We wanted to see if this is also the case for a developing country like Romania [8], where corporate volunteering is sometimes seen as a practice that is used by some companies as way to avoid paying employees for their work. Thus, we focused on researching the impact of management support for volunteering on organizational environmental volunteering and of the latter on employees’ well-being and productivity.

Other studies emphasize the risks posed by organizational volunteering, as employees might experience burnout and feel overwhelmed by the prospect of engaging in volunteering commitments alongside their daily work tasks [9]. Therefore, management’s role is essential in shaping adequate CSR strategies for employees and ensuring a good work–life balance because volunteering is still work, even if unpaid.

In Section 2 of the paper, we focus on recent studies about the four variables included in our model for Romanian organizational volunteering; further, we present a research methodology based on the use of partial least squares structural equation modeling and the four hypotheses we develop; we continue with Section 4, in which we present the results of our research and test the hypotheses; in the Discussion, section we analyze each hypothesis and present other authors’ findings; and in the Conclusions, we focus on the main theoretical and managerial implications of our research, but also on its limitations and future research directions.

## 2. Literature Review

Air pollution poses significant health risks, affecting every country in the world. This issue was addressed by numerous studies that emphasize the importance of citizen initiatives in tackling this stringent problem [10,11,12]. Considering the role of environmental volunteering in promoting green behaviors in the community, this literature review allows us to identify four variables that connect organizational environmental volunteering with well-being and productivity and the support received by the employees from their managers. CSR activities oriented towards employees might also take the form of corporate volunteering, meaning that the company benefits from a better image in the community and this may influence its relationship with its business partners. A ripple effect might also appear and motivate other businesses to implement similar approaches [13].

### 2.1. The Management Support for Organizational Volunteering

As other authors showed [9], organizational volunteering has both advantages and disadvantages, and managers should carefully consider the time the employees spend working on their daily tasks and the time they dedicate to volunteering to avoid burnout or other harmful issues. Management support might take various forms, from simple encouragement to praising volunteers or offering flexible work arrangements to balance their activities. MacPhail and Bowles [14] noticed an essential difference between the support offered to men and women in Canada. Their findings show that women “are less likely to receive support in the form of flexible work hours and time-off” compared to men [14] (p. 405). Peloza et al. [15] (p. 371) studied nine large companies from “a Midwestern city” and concluded that “manager recognition or time off is not effective”. Therefore, the context (in other words, the dimension of the company or the city/country under analysis) is essential. Different authors might reach different conclusions because how people perceive volunteering and how managers support them depends on many specific factors. This is one of the reasons we focused on Romania, a developing country with potential for volunteering, because of the low number of volunteers compared with those in other, more developed countries [8]. A more general approach that does not consider the specifics of each country and culture might not be appropriate or relevant for shaping adequate CSR and human resources strategies. Gatignon-Turnau and Mignonac [16] highlight that company support of organizational volunteering is not always followed by increased volunteering activity among employees because there is also a risk that they will perceive the CSR program as a selfish one that is meant to improve the company’s image and benefits. Zappala and McLaren [17] (p. 52) emphasize that some employees might attend volunteering programs “because of a perception that they were expected to,” but there are no “feelings of resentment” afterward.

### 2.2. Organizational Environmental Volunteering

Many benefits are associated with employees’ engagement in organizational environmental volunteering, from the satisfaction associated with contributing to a cleaner environment to more personal benefits related to the communication skills they gain or the social interactions they develop. Peterson [5] found that employees who volunteer are more committed to their organizations and focus on gaining skills they can use at their jobs. Another perceived benefit was the satisfaction felt, which Peterson [5] noticed only among the women in the study. There was also a difference between persuading employees to engage in a volunteering activity on one occasion and maintaining their interest during several CSR projects developed by the organization [18]. Peterson [18] noticed that age is also an important factor when the employees choose one volunteering program or another: youngsters are more oriented toward their career goals, while older employees are also interested in the social part of the program. Organizational volunteering is a broader term for corporate volunteering and includes all sorts of organizations, encompassing not only corporations but also public institutions. Sanchez-Hernandez and Gallardo-Vázquez [19] (p. 397) state that there more higher chances for a company to achieve its CSR objectives if there are programs of organizational volunteering for employees, and also that these initiatives “strengthen the bonds between the company and its employees”. Boštjančič et al. [20] mention that volunteering has psychological benefits and the volunteers are “more engaged and report higher levels of both autonomy and support”. Herzig [21] (p. 51) highlights that organizational volunteering is mainly specific to “larger and multinational enterprises” in Germany. Big companies can easily develop CSR initiatives for important projects related to fighting climate change because they have more financial resources and experience than small- and medium-sized enterprises.

### 2.3. Employees’ Well-Being

Page and Vella-Brodrick [22] (p. 441) appreciate that there are three aspects of well-being: “subjective well-being; … workplace well-being and … psychological well-being”. The authors analyzed the role played by the employees’ well-being in terms of the organization’s performance and in minimizing personnel turnover. Harter et al. [23] (p. 205) mention the benefits of well-being among employees: “customer loyalty, higher profitability, higher productivity, and lower rates of turnover”. Pawar [24] (p. 975) highlights the following types of well-being: “emotional, psychological, social, and spiritual well-being”. Several authors [24,25] consider that developing spirituality at the organizational level positively influences employees’ well-being. Wright and Huang [26] (p. 1188) mention “job performance, employee retention” as benefits for enhancing well-being. Guest [27] emphasizes that human resources managers should consider ways to improve employees’ well-being, gravitating away from the traditional perspective of focusing solely on their performance. As other authors concluded [22,23,26], performance is influenced by well-being at work, so acknowledging this causal relationship helps shape better CSR and human resources strategies oriented toward the organization’s employees. Ter Hoeven and Van Zoonen [28] appreciate that flexible work arrangements are one way to raise the level of well-being at work. Still, there are also downsides related to interruptions during working time. Therefore, managers should consider both the positive and negative impact that greater flexibility can have on employees’ well-being.

### 2.4. Employees’ Productivity

Many factors positively or negatively influence employees’ productivity at work. Thus, investments in training for developing specific skills required for the job are needed to increase productivity [29,30], but it is also necessary to encourage employees to participate in decision-making within the organization [31]. Bhatti and Qureshi [31] conclude that employees’ participation positively impacts both productivity and satisfaction. Mitchell et al. [32] found that employees’ productivity is also raised by their participation in health programs that are meant to improve their health. Thus, employees will take fewer days off due to medical leave, and the organization will save money that would otherwise have been lost [32]. Holzer [33] states that there is a positive correlation between experience and both productivity and wage level, but also notices differences between men and women: the women registered a higher increase in their productivity levels while having lower wages than men. Joo and Grable [34] identified that providing financial counseling to their employees to help them better manage their money also increased their productivity because they became less preoccupied with their problems. These findings show us that employees’ personal problems affect them at work. Managers should communicate frequently with their subordinates to find a solution and not let problems escalate to the detriment of both the organization and the employee.

## 3. Research Methodology

The main objective of our research is to better understand how environmental volunteering organized by companies and institutions influences the well-being and productivity of employees. We appreciate that these aspects are important in the broader context of climate change and increased pollution in the world, which affects us all [5,7]. We used partial least squares structural equation modeling and the software SmartPLS, version 4 [35] as the research methodology. After reviewing the literature on environmental volunteering, we developed the following hypotheses that consider its impact on well-being and productivity with the goal of understanding the situation in Romania, a developing country with low levels of volunteering [8]:

**Hypothesis 1** **(H1):** *Management support has a direct and positive impact on the organizational environmental volunteering of employees.*

**Hypothesis 2** **(H2):** *The organizational environmental volunteering of employees has a direct and positive impact on their well-being.*

**Hypothesis 3** **(H3):** *The organizational environmental volunteering of employees has a direct and positive impact on their productivity.*

**Hypothesis 4** **(H4):** *Employees’ well-being directly and positively impacts their productivity.*

**Figure 1:**
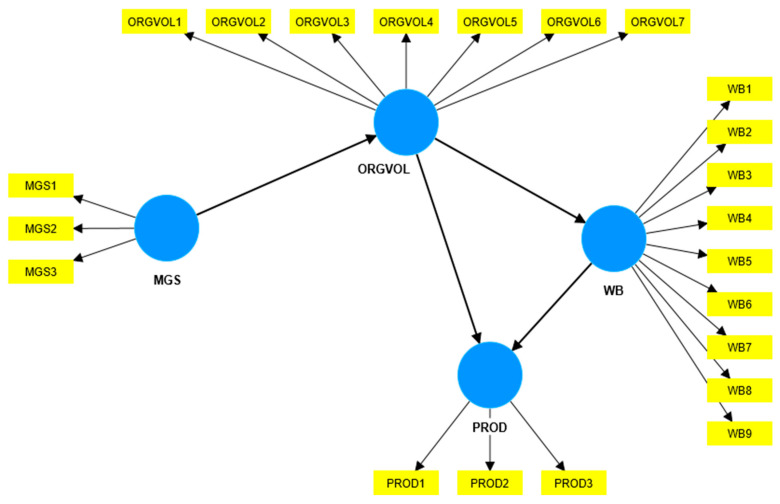
The conceptual model, created by authors with SmartPLS, version 4.

To test these hypotheses and achieve the objective of our research, we propose the conceptual model in Figure 1. The model considers four variables, which are as follows: management support for organizational environmental volunteering (MGS1–MGS3), organizational environmental volunteering (ORGVOL1–ORGVOL7), employees’ well-being (WB1–WB9), and employees’ productivity (PROD1–PROD2).

Table 1 presents the model’s variables, the items, and their codes.

The data were collected using a questionnaire created with Google Forms, which was distributed in Romania in August and September 2023 to 600 employees engaged in organizational environmental volunteering in fields like the automotive industry, telecommunication, education, energy, and IT. We used the non-probabilistic snowball sampling method. We obtained 213 valid answers to our survey, which surpassed the minimum number required for using SmartPLS [36]. A total of 84.5% of the respondents had completed higher education, 54.5% had worked for their organizations for more than ten years, and 63.4% were between 36 and 55 years old. To apply the PLS-SEM, the statements in the survey (also included in Table 1) were built using a Likert scale from 1 to 5, where 1 is total disagreement, and 5 is total agreement. The questionnaire included demographic questions related to age, studies, and work experience and 22 questions related to the four variables addressed: three questions about management support, seven about organizational environmental volunteering, nine questions on well-being, and three on productivity. The studies used to build the questionnaire are presented in Table 1.

## 4. Results

To test the reliability of the items in our model, we needed to determine the outer loadings. Thus, in Table 2, we present the outer loadings and variance inflation factor (VIF) for the items in the research model using the PLS-SEM algorithm. Both show the items’ convergent validity because all the outer loadings are above 0.5 and the VIF values are under 4. Most items (19 out of 22) have outer loadings over 0.7, which is an indicator of their high reliability [37], but the values between 0.5 and 0.7 for the other three items are considered acceptable [38].

**Figure 2:**
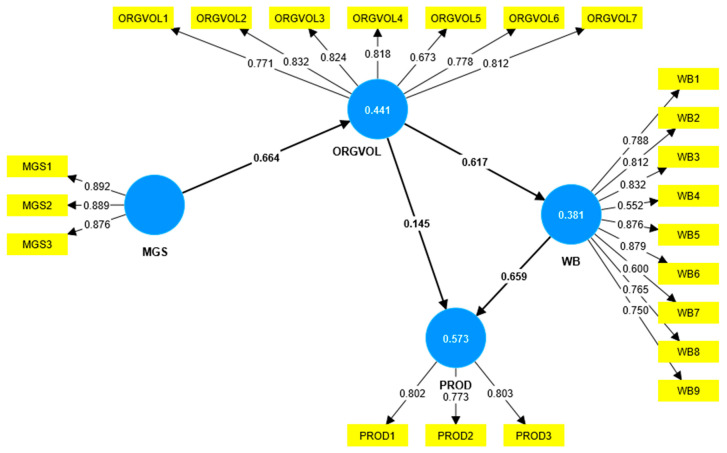
PLS-SEM algorithm calculated with SmartPLS, version 4.

In Figure 2, we illustrate the model including the values for the outer loadings and the impact of each construct on the others. We notice that the most substantial impact is from MGS to ORGVOL (0.664), followed closely by the impact from WB to PROD (0.659) and from ORGVOL to WB (0.617). MGS is responsible for 44.1% of the ORGVOL variance; ORGVOL is responsible for 38.1% of the WB variance; ORGVOL and WB are responsible for 57.3% of the PROD variance.

The construct reliability and validity of the model are checked in Table 3 using SmartPLS [32].

Cronbach’s alpha and composite reliability are above 0.7, and the average variance extracted is above 0.5, which shows the high reliability and validity of the research model we proposed [39]. To measure the model’s discriminant validity, we applied the Fornell–Larcker criterion, as shown in Table 4.

The values in the main diagonal are higher than the ones relating each construct in the model to the others, which shows that they are different enough to make the model valid. Further, we applied the bootstrapping test in Table 5 to see if the model is statistically significant.

All t statistics are above 1.96, all p values are below 0.05, and the bias-corrected confidence intervals do not include zero, so the model is considered to be statistically significant, and all four hypotheses are validated.

Table 6 presents the mean and the standard deviation for the items in the model. Most of the means are above four (corresponding to the agreement on the Likert scale), with the lowest registered for support received from the management, especially regarding training or praising the volunteers during internal e-mails or official meetings. The other means show that the employees perceive themselves as happy and satisfied (exhibiting a high level of well-being) and having high productivity at work.

Regarding their motivations for engaging in volunteering activities, most respondents (65.1%) mentioned the feeling of being able to help the community. They were followed by those who mentioned that volunteering matches their values (37.1%), helps them with communication skills (36.6%), and offers the possibility of meeting new people (34.9%). All these motivations have a social component that is part of well-being.

## 5. Discussion

Further, we present the conclusions reached by other authors about the hypotheses we developed and the four variables included in our model: management support for employee volunteering, organizational environmental volunteering, and employees’ well-being and productivity.

Hypothesis H1: Management support has a direct and positive impact on the organizational environmental volunteering of employees. This hypothesis was validated, showing that managers can influence employees’ decision to engage in environmental volunteering if they encourage them, praise them, or debate environmental problems and volunteering during training. The impact highlighted by this hypothesis is also the strongest (0.664), as shown in Figure 2. Al Kerdawy [40] showed that management support for volunteering is an indicator of developing CSR activities and Geroy et al. [41] (p. 280) state that employees who volunteer “reap high overall rewards” if they have their supervisors’ support. Cycyota et al. [42] conducted research on 100 organizations in the Fortune rank of the best companies to work for, emphasizing that these businesses value volunteering among their employees by encouraging them and integrating these activities into their CSR strategy. Basil et al. [43] show that the size of the company also matters when we refer to the support of management for corporate volunteering because big companies are more prone to engaging in these activities and have official strategies and policies related to volunteering (as part of CSR programs) than smaller ones, which can be explained through the lens of experience and also financial security. When companies show support of volunteering, such as by offering free days or more flexible work arrangements for their employees, these actions are often perceived by organizations as a way to improve their image in the community and thus, as part of their CSR [44].

Hypothesis H2: The organizational environmental volunteering of employees has a direct and positive impact on their well-being. This hypothesis was validated, showing that there is a strong correlation (0.617) between these two variables in the model (ORGVOL and WB). Employees involved in volunteering activities register higher levels of happiness and satisfaction at work. Binder and Freytag [45] highlight the correlation between regular volunteering and well-being, which can be useful for shaping public policies. Piliavin and Siegl [46] (p. 450) emphasize that “consistency of volunteering over time and diversity of participation” are important to the significance of its impact on well-being. Brown et al. [47] (p. 468) concluded that “volunteers will report higher well-being than nonvolunteers”, but also “self-esteem, self-efficacy, and social connectedness”. Zhang et al. [48] noticed a correlation between the “employee green behavior” and their well-being, a relation which was moderated by the support they received from their managers. These findings are useful for companies, as they can integrate these green activities into their strategies and thus reduce their carbon footprint and impact on the environment. Son and Wilson [49] conducted research on volunteers (not specifically on employees) and reached the conclusion that volunteering influences the social part of well-being no matter how many hours people volunteer for. Considering these findings, it is useful for managers to encourage their employees to volunteer to strengthen the bond between colleagues and thus reduce potential work conflicts.

Hypothesis H3: The organizational environmental volunteering of employees has a direct and positive impact on their productivity. This hypothesis was validated, showing that volunteers are also more productive at work, at least according to the employees’ perception. Still, when questioned about the internal evaluations (performed by their managers), 93.4% of the respondents said that these employees’ productivity was good (29.1%) or very good (64.3%), so the employees’ perceptions of their own productivity are probably close to those of their supervisors. Knox [50] (p. 449) found that there was a positive relationship between corporate volunteering and employees’ productivity “up to 6 years after a firm uses an employee volunteer program”. Brenner [51] appreciates the role played by volunteering in raising the level of work productivity but also of the profitability of a company.

Hypothesis H4: Employees’ well-being directly and positively impacts their productivity. This hypothesis was validated, which is also emphasized by the strong impact from WB to PROD (0.659), as we can see in Figure 2. This means that employees with a great level of happiness and satisfaction at work (which also pertains to their relationships with their colleagues and supervisors) are also more productive. Haddon [52] emphasizes the role of well-being in an organization’s performance. The author states that a low level of well-being negatively influences both the employee and his/her colleagues, causing a deterioration in the work climate. Many authors [53,54] identified the positive impact of employees’ well-being on their work productivity, highlighting the need to adjust human resources policies in a way that promotes satisfaction and happiness among employees.

## 6. Conclusions

The main objective of our research was to identify the connection between environmental volunteering of employees and the impact on their well-being and productivity, while understanding the role played by management in encouraging these CSR initiatives, which can contribute to a more sustainable environment, less pollution, and a world in which renewable energy is used more and more.

### 6.1. Theoretical and Practical Implications

Our research on organizational environmental volunteering and the impact it has on both employees’ well-being and productivity is important for both researchers and for managers of organizations. This is especially relevant for a developing country like Romania where volunteering is not always viewed positively [55]. Our literature review is rich in papers focusing on topics that are related to CSR, and sustainability but also with the SDGs in the 2030 Agenda 2030 of the United Nations (especially SDG 3—Good Health and Well-Being, SDG 8—Decent Work and Economic Growth and SDG 13—Climate Action).

Our findings are valuable for managers in both public and private organizations, as they will help them to build CSR strategies meant to raise the level of happiness, job satisfaction, and performance of their employees. CSR in the form of organizational volunteering opportunities for employees can be also a human resources strategy for attracting people with specific qualities, and ensuring their retention in the organization. Still, the benefits are not only targeted toward the employees but also the company’s image and financial results because there is a strong relationship between the employees’ well-being, their productivity [50], and the profitability of the organization.

### 6.2. Limitations of the Research and Future Research Directions

The limitations of our research were related to collecting the data online and using social media channels, mainly Facebook, to reach the employees who are engaged in organizational volunteering. Another limitation was related to the fact that volunteering is less common in Romania than in other European countries because some people might perceive organizational volunteering as a way for the company to use their resources and not pay them for their work [8]. Considering these aspects, especially for Romania, a developing country, it was difficult to reach a reasonable number of volunteers to apply the PLS-SEM method. For future research directions, we will consider taking into account the impact of organizational environmental volunteering on the relationship with the company’s customers, the role of management styles in employees’ motivation to engage in volunteering, and the relationship between volunteering and work climate, but also the link to burnout. Other research directions can focus on managers’ perspective of environmental volunteering and its impact on organizational profitability and long-term image in the community. As things have started to move in a good direction in Romania, continuous research in this area might provide different results. Since 2014, there has been a law [56] dedicated to volunteering that ensures these activities are recognized as work experience if they are in the field of graduate studies.

## Figures and Tables

**Table 1:** The variables, items, and codes of the proposed model.

Variables	Items	Codes
Management support for environmental volunteering (MGS) [14,15,16,17]	The organization’s managers encourage employees’ involvement in volunteering activities.	MGS1
	The organization provides training for its employees related to environmental volunteering.	MGS2
	The managers in the organization praise the employees who are engaged in volunteering.	MGS3
Organizational Environmental Volunteering (ORGVOL) [5,19,21]	I engage in volunteering activities because my family and friends motivate me.	ORGVOL1
	I engage in volunteering activities because my manager also volunteers.	ORGVOL2
	I engage in volunteering activities because my colleagues also volunteer.	ORGVOL3
	I engage in volunteering activities because it allows me to learn new things.	ORGVOL4
	I engage in volunteering activities because I can help someone.	ORGVOL5
	I engage in volunteering activities because they impact the local communities.	ORGVOL6
	I engage in volunteering activities because they have an impact at the national level.	ORGVOL7
Employees’ well-being (WB) [22,24,25,26]	My workplace is a source of satisfaction for me.	WB1
	I enjoy what I do at my workplace.	WB2
	I feel happy at my workplace.	WB3
	I like to interact with people.	WB4
	I am proud to have worked for this organization.	WB5
	I believe in my organization’s mission.	WB6
	The quality of the relationships I have with my colleagues is high.	WB7
	The quality of the relationships I have with my supervisors is high.	WB8
	I have the opportunity to develop myself in the organization where I work.	WB9
Employees’ productivity (PROD) [31,32]	My productivity at work is high.	PROD1
	I always try to do more than the minimum required at work.	PROD2
	I gained new abilities at my workplace.	PROD3

**Table 2:** Outer loadings and VIF for the items in the research model.

Items	Outer Loadings	VIF
MGS1	0.892	2.046
MGS2	0.889	2.380
MGS3	0.876	2.293
ORGVOL1	0.771	1.935
ORGVOL2	0.832	3.789
ORGVOL3	0.824	3.657
ORGVOL4	0.818	2.450
ORGVOL5	0.673	1.657
ORGVOL6	0.778	2.695
ORGVOL7	0.812	3.034
WB1	0.788	2.989
WB2	0.812	3.395
WB3	0.832	3.781
WB4	0.552	1.355
WB5	0.876	3.932
WB6	0.879	3.706
WB7	0.600	1.882
WB8	0.765	2.385
WB9	0.750	2.151
PROD1	0.802	1.585
PROD2	0.773	1.562
PROD3	0.803	1.237

**Table 3:** Construct reliability and validity.

Constructs	Cronbach’s Alpha	Composite Reliability (rho_a)	Composite Reliability (rho_c)	Average Variance Extracted (AVE)
MGS	0.863	0.876	0.916	0.784
ORGVOL	0.898	0.906	0.920	0.622
PROD	0.711	0.726	0.835	0.628
WB	0.910	0.918	0.928	0.592

**Table 4:** Fornell–Larcker criterion applied to the model.

Constructs	MGS	ORGVOL	PROD	WB
MGS	0.886			
ORGVOL	0.664	0.789		
PROD	0.384	0.552	0.793	
WB	0.576	0.617	0.748	0.769

**Table 5:** Bootstrapping test.

Constructs	T Statistics	*p* Values	Confidence Intervals Bias Corrected	Hypotheses Validation
MGS → ORGVOL	16.858	0.000	(0.575, 0.733)	H1 validated
ORGVOL → WB	11.678	0.000	(0.494, 0.708)	H2 validated
ORGVOL → PROD	2.467	0.014	(0.027, 0.256)	H3 validated
WB → PROD	11.022	0.000	(0.523, 0.760)	H4 validated

**Table 6:** The descriptive statistics for the items in the model.

Items	Mean	Standard Deviation
MGS1	3.718	1.338
MGS2	3.023	1.533
MGS3	3.178	1.449
ORGVOL1	3.728	1.271
ORGVOL2	3.521	1.416
ORGVOL3	3.737	1.324
ORGVOL4	4.230	1.074
ORGVOL5	4.479	0.864
ORGVOL6	4.009	1.088
ORGVOL7	3.878	1.132
WB1	3.991	1.057
WB2	4.362	0.896
WB3	4.056	0.987
WB4	4.638	0.689
WB5	4.254	0.994
WB6	4.225	1.042
WB7	4.385	0.800
WB8	4.164	0.953
WB9	3.977	1.265
PROD1	4.333	0.797
PROD2	4.545	0.759
PROD3	4.362	0.912

## Data Availability

Available upon written request.
